# Factors associated with crisis pregnancies in Ireland: findings from three nationally representative sexual health surveys

**DOI:** 10.1186/s12978-015-0005-z

**Published:** 2015-03-02

**Authors:** Ashling Bourke, Caroline Kelleher, Daniel Boduszek, Karen Morgan

**Affiliations:** Division of Population Health Sciences, Royal College of Surgeons in Ireland, 123 St. Stephen’s Green, Dublin, 2 Ireland; Education Department, St Patrick’s College, Drumcondra, Dublin, 9 Ireland; Department of Behavioural and Social Sciences, University of Huddersfield, Room R2/23, Huddersfield, HD1 3DH UK; Perdana University-Royal College of Surgeons in Ireland School of Medicine, Perdana University, 43400 Serdang, Darul Ehsan Malaysia

**Keywords:** Crisis pregnancy, Unintended pregnancy, Sexual health, Sex education, Abortion, Ireland

## Abstract

**Background:**

Findings on the demographic and sexual health characteristics associated with the experience of a crisis pregnancy are important to inform the public health policy of a country, including Ireland. Studies from other jurisdictions have suggested that certain demographic groups are at risk for unintended pregnancies and the disparity between the groups has been growing in recent years. Ireland is a country which experienced much economic and societal change in the first decade of the 21^st^ century; changes which are likely to have affected demographic variables pertaining to sexual health. The current study had two aims: to investigate changes in the socioeconomic characteristics associated with crisis pregnancies over a seven year period [2003 to 2010], and to investigate the recent [2010] socioeconomic risk factors associated with crisis pregnancies in Ireland.

**Methods:**

The study compared the results from 18–45 year old women using data from three broadly similar nationally representative Irish sexual health surveys carried out in 2003, 2004–2006 and 2010. Chi square analysis compared of the socioeconomic characteristics across the seven year period. A logistic regression then investigated the sexual health history and socioeconomic factors associated with the experience of a recent crisis pregnancy using the most recent 2010 data.

**Results:**

In 2010, 74% of women experienced parenthood and 23% experienced abortion as the outcome of their crisis pregnancy. Receipt of sex education and contraception use at first sex significantly predicted the experiencing of a recent crisis pregnancy. Younger women and those with a lower level of education were more likely to report having experienced a recent crisis pregnancy.

**Conclusion:**

Similar demographic groups are at risk for experiencing a crisis pregnancy in Ireland compared with international research, yet the disparities between demographic groups who have experienced a crisis pregnancy appear to be decreasing rather than increasing over a seven year period. Recommendations are made with regard to the provision of continued sex education throughout the lifespan, particularly for those women who are at an increased risk of experiencing a crisis pregnancy.

## Background

Approximately 80 million unintended pregnancies occur globally each year, of which 42 million result in induced abortions and 34 million resulted in unintended births [1]. The adverse consequences associated with unintended pregnancies include low birth weight, preterm birth, less prenatal care and increased likelihood of post-partum depression and psychological distress [2-5]. Given these negative outcomes, and in order to inform prevention strategies, research has largely focused on the prevalence and causes of such pregnancies, while also trying to identify the demographic groups most at risk for these pregnancies nationally [6,7]. The current study aims to investigate these factors in Ireland.

A number of different terms have been used throughout the literature to refer to these pregnancies, such as unplanned and unintended pregnancy, indicating the pregnancy was mistimed [8,9]. However, although a pregnancy may be unplanned or mistimed, it may also be a welcome surprise for those involved. In order to overcome some of the limitations in the definition of such pregnancies, the term crisis pregnancy is used in Ireland to identify those pregnancies that represent a personal crisis or trauma for the woman or couple involved. This trauma is subjective and may be experienced at any point during the pregnancy; a pregnancy is defined as a crisis pregnancy if it began as a crisis even if the crisis was subsequently resolved, or if a pregnancy develops into a crisis before the birth due to a change in circumstances [10]. In Ireland, the most common reason given (by 41% of the women) for a pregnancy being described as a crisis pregnancy was that it was not planned [10]. Further reasons given for a pregnancy being defined as a crisis differed according to the outcome of the pregnancy, such that for women who chose to parent, being too young and not married were common reasons given, whereas for women whose pregnancies resulted in abortions, the crisis was due to relationship difficulties, not wanting the baby, or being too young [10]. As these reasons suggest, less than half of the reasons given for a crisis pregnancy involved the planning of the pregnancy, and many of the reasons were due to life circumstances. This limits and complicates any comparisons with other jurisdictions where the intendedness of a pregnancy is assessed. Drawing on these findings, one must be cautious when comparing Irish data to unintended pregnancy data from other jurisdictions, as the Irish data is looking at a much broader variable, and thus the prevalence rates of crisis pregnancies are likely to be higher than that of unintended or unplanned pregnancies.

A recent national report from Ireland found that 16% of all pregnancies experienced by women in 2010 were defined as a crisis pregnancy [5]. However, there are only a small number of studies in Ireland on this topic, and therefore much of the research cited here comes from the US. Although there are likely to be similarities between the two countries, differences such as healthcare provision, wealth distribution, the role of religion in society, and most notably the type of pregnancy included in the research, should be borne in mind when interpreting these findings. The United States has one of the highest rates of unintended pregnancy in the developed world, where it has been estimated around 37% of all births [8] and 49% of all pregnancies [6,11] (an equivalent of 5.2% of the population of 15 to 44 year old women [6]), are unintended. Furthermore, although the prevalence rate for unintended pregnancy in the US has remained relatively stable over the past decade, the disparities between the demographic groups who experience an unintended pregnancy have increased [6]. The demographic risk factors for unintended pregnancy in the US include: younger age [6,5,12,13]; lower levels of education [6,13]; lower income [6,14]; cohabiting relationship status [6]; and one previous birth [6]. The increase in the disparities between the groups is reflected in the finding that although the rate of unintended pregnancies fell among higher income women in the US between 2001 and 2006, the rate of unintended pregnancies among poor, low income women increased substantially [6].

The primary cited causes of unintended pregnancies include, a lack of contraception use [8,14,15]; use of ineffective contraception methods [16]; contraceptive failure [16]; and the incorrect or inconsistent use of effective contraceptive methods [14,16,17]. Irish qualitative research found that most women found dealing with sex and contraception stressful and problematic and almost all respondents reported having unsafe sex at some stage [18]. Cost has been cited as a barrier to accessing both condoms and prescription contraceptives [5,19]. In Ireland, prescription contraceptives carry the cost of doctor consultation, administration and/or prescription costs. These costs may be covered by free medical care through the medical card system for those on a low income. Any individual who is ordinarily resident in Ireland (someone who has lived in Ireland for at least one year or intends to live in Ireland for at least one year) is entitled to apply for a state medical card. Condoms are not available on medical cards. A number of sexual health behaviours have also been associated with contraception use. For example, early age of first sex has been associated with gaps in contraception use in adulthood [20]. Additionally, sex education has been associated with contraception use at first sex [21,22] and a lower level of teenage pregnancies [23]. However, relatively little attention has been given to the association between sexual health histories, such as sex education and the context of first sex, and the incidence of unintended or crisis pregnancies.

The outcomes of unintended and crisis pregnancies are also of interest to researchers. Globally, the rate of giving birth, when faced with an unintended pregnancy, is lower than that of induced abortions [1]. However, differences in abortion legislation across jurisdictions are likely to affect this outcome. In the US, there has been a slight decline in the number of unintended pregnancies ending in abortion between 2001 (47%) and 2006 (43%) [6]. In Ireland, recent changes to legislation, followed from the death of a woman from septicaemia after she was refused an abortion, allows for an abortion in the state only if the woman’s life is physically at risk. A recent national survey in Ireland found that of those women, aged 18 to 45 years, who ever experienced a crisis pregnancy, 73% experienced parenthood and 24% experienced abortion as the outcome of their pregnancy [5].

The current study aims to investigate any socioeconomic inequalities prevalent in the experience of crisis pregnancy in Ireland and how these may have changed over the past number of years. Ireland has undergone significant changes economically and demographically over the past decade. These social and economic changes have implications for sexual health practices that may be sensitive to the changing make-up of the population. By comparing data from three sexual health surveys carried out at different time points across the past decade in Ireland, the current study will;

- Identify whether the demographic characteristics of those women who have experienced a recent crisis pregnancy has changed over a seven year period (from 2003 to 2010)
- Examine the change in the individual’s age, contraception use, and outcomes of crisis pregnancies over the same period (from 2003 to 2010)
- Generate a profile of the socio-demographic characteristics and the early sexual experiences of women who experience a crisis pregnancy in Ireland, using the data from the most recent survey in 2010.

## Method

### Surveys

Data were obtained from three nationally representative cross-sectional sexual health surveys, carried out in Ireland between 2003 and 2010: the Irish Contraception and Crisis Pregnancy Survey 2003 (ICCP-2003) [10] conducted between 2003 and 2004 (n = 3317; age range 18–45 years); the Irish Study of Sexual Health and Relationships (ISSHR) [19] conducted between 2004 and 2006 (n = 7441) (age range 18–64 years); and Irish Contraception and Crisis Pregnancy Survey 2010 (ICCP-2010) [5] conducted in 2010 (n = 3002) (age range 18–45 years). Variables common to all three surveys were selected for investigation. Any inter-survey differences in these variables are highlighted where necessary. Ethical review of the three surveys was done by the Research Ethics Committee of the Royal College of Surgeons in Ireland. The aims of the surveys were to profile the beliefs, attitudes and behaviours related to sex, contraception and pregnancy of adults living in Ireland. In the 2003 and 2006 surveys, respondents were interviewed using landline telephones numbers, whereas in the 2010 survey, respondents were interviewed using both landline and mobile telephones, to reflect the decline in landline ownership and increase in mobile phone use. Calls were made using Random Digit Dialling (RDD). The overall response rate was 64% in 2003; 61% in 2006 and 69% in 2010. Detailed methodologies for each survey are available elsewhere [10,19,5].

### Crisis pregnancy variables

The question pertaining to a crisis pregnancy was asked as follows: ‘Now I would like to ask you about what we would describe as crisis pregnancies. By this I mean a pregnancy that represents a personal crisis or emotional trauma. This can be a pregnancy that began as a crisis but over time the crisis was resolved. It can also include a pregnancy which develops into a crisis before the birth due to a change in circumstances’ [10,19,5]. Respondents were asked their age at the time of the crisis pregnancy (coded as 15–19 years; 20–24 years; 25–29 years; 30–34 years; 35 years and older); whether any contraception was used and the method used at the time of conception (coded as ‘no contraception’, ‘low efficacy contraception’, ‘medium efficacy contraception’ or ‘high efficacy contraception’, according to the Pearl Index: the Pearl index is a common technique used to determine the efficacy of contraceptive methods and calculates the frequency of pregnancies that occur over a specific time period per method of contraception); the year the pregnancy occurred; and the outcome of their crisis pregnancy (coded as parenthood or abortion) (adoption was not included as an outcome given its low frequency (less than 1.2%)). For those respondents who reported more than one crisis pregnancy, they were asked to provide information on their most recent crisis pregnancy only. If more than one contraception type was reported, it was coded according to the method with the highest efficacy.

### Respondent characteristics

Respondents were asked a number of demographic questions, including their current relationship status (coded as married (and living with spouse); cohabiting; steady relationship and not cohabiting; and casual or no relationship) and their highest level of education (coded as pre-Leaving Certificate; Leaving Certificate; and post Leaving Certificate). Leaving Certificate corresponds to completed second level education in Ireland. Respondents were asked question regarding importance of religious beliefs. In both the ICCP-2003 and ICCP-2010 surveys, respondents were asked ‘How important are your religious beliefs to you’ on a five point Likert scale from 1 (‘very important’) to 5 (‘not at all important’). In the ISSHR survey, respondents were asked ‘Would you describe yourself as a religious or spiritual person’ on a five point Likert scale that ranged from 1 (‘not at all’) to 5 (‘extremely’). This religiosity variable was recoded ‘important’ or ‘not important’. Respondents were also asked where they were born and the age they moved to the Republic of Ireland (ROI) if they were not born there. Respondents who moved to ROI aged 16 years or over were coded as ‘migrant after 16’ and those who migrated at a younger age or were born in the ROI were coded as ‘not a migrant after 16’. Respondents were asked if they were in receipt of free medical care. There are two categories of state subsidised medical care in Ireland; the medical card which covers General Practitioner (GP) consultations and prescriptions, and GP only medical cards which covers GP consultations only. This was coded as ‘full medical card’, ‘GP only card’, and ‘no medical card’. Data relating to respondents occupation and that of the head of the household were coded according to the Central Statistics Office (Ireland) guidelines. The data were coded similarly across the three surveys; however, in ICCP-2003 and ISSHR those respondents who were currently unemployed were coded according to their most recent employment, whereas in ICCP-2010 those respondents who reported unemployment were not asked about their previous employment. This variable was coded as SC 1–2 including professional workers and managerial and technical workers (reference category); SC 3–4 including non-manual and skilled manual workers; SC 5–6 including semi-skilled and unskilled workers; and SC 7 which included all others, including never worked and long-term unemployed. Each of the above variables are presented as ‘current’ status, such that they were taken at the time of the survey rather than at the time of the crisis pregnancy.

### Sexual health history

Variables pertaining to the respondents’ sexual health history were also obtained from the most recent survey, ICCP-2010. All respondents were asked whether they received sex education growing up (between 10–16 years) (coded as ‘receipt of sex education’). Data from respondents who reported ever having experienced heterosexual intercourse was obtained in terms of the age of first sex (dichotomised as occurring before or after the legal age of consent in Ireland, currently 17 years of age). Respondents were also asked whether they used contraception on this occasion (coded as ‘yes’ or ‘no’).

### Data inclusion and weighting

Only female respondents, aged between 18 and 45 years were included in the analysis (ICCP-2003 n = 1961; ISSHR (2006) n = 2979; ICCP-2010 n = 1562). Those respondents who experienced a crisis pregnancy more than six years prior to the survey were not included in the main analysis. The cut-off of six years was chosen for a number of reasons: to minimise the overlap between the time periods being assessed for each of the three surveys; to balance the effects of inaccurate recall over a longer period of time while ensuring an adequate sample size; to minimize the effect of the demographic characteristics of the respondents changing since the experience of a crisis pregnancy. Quota sampling was used to ensure the sample was representative of the general population at the time of the surveys. All three studies used a minimum distance algorithm to implement the reweighting adjustment to ensure the data were fully representative of the Irish population at the time of the survey.

### Analytic plan

The analysis was carried out in three steps. First, the demographic characteristics of those who experienced a recent crisis pregnancy were compared with the overall sample across the three surveys. Chi squared analyses examined whether there were any differences on these demographic variables across the three surveys in terms of who experienced a recent crisis pregnancy. Second, the characteristics of those respondents who experienced a recent crisis pregnancy was examined across the three surveys, specifically their age at the time of the crisis pregnancy, the contraception used at the time of conception and the outcome of the crisis pregnancy (parenthood or abortion). Chi square analyses were conducted to investigate any significant differences on these variables across the three surveys. These chi square analyses were conducted in unweighted data. Given the number of analyses that were carried out a Bonferonni correction was applied, with a resultant alpha level of 0.005. Third, two hierarchical logistic regressions were conducted. These two regressions examined the sexual health history and current demographic profile of respondents who experienced a crisis pregnancy. The first regression investigated the profile of those who experience a crisis pregnancy in the six years immediately prior to 2010 (n = 1273), using weighted data. A second regression analysis was conducted for individuals who had *ever* experienced a crisis pregnancy. The second regression results are presented briefly to highlight the differences in predictors of the experience of a crisis pregnancy recently and the experience of a crisis pregnancy ever. Analyses were carried out on SPSS 20.0 [24].

## Results

### Demographic characteristics of women who experienced a recent crisis pregnancy [2003 – 2010]

Table 1 presents the percentage of those in each of the demographic groups who experienced a recent crisis pregnancy compared with the overall survey group, across each of the three surveys. The percentage of women in the older age category (34–45 years) who experienced a recent crisis pregnancy increased from 3.5% in 2003 to 5.6% in 2010, but this difference was not significant. There was a significant difference found in the social class of those respondents who experienced a recent crisis pregnancy across the survey years. A higher proportion of respondents from SC7 (the employees who were unclassified and the unemployed) reported having a crisis pregnancy in 2010 compared to 2006. There was also a significant effect found for education level; compared with other levels of education, a higher proportion of respondents with Leaving Cert, compared with post-Leaving Cert level of education reported a crisis pregnancy in 2006. Descriptive statistics indicate that the percentage of women with pre-Leaving Certificate education who experienced a recent crisis pregnancy has increased from 2003 (9.5%) to 2010 (13.6%). A higher proportion of women with two or more children experienced a crisis pregnancy in 2010 compared with 2006. There was no statistical difference found across the surveys in terms of the relationship status of those who experienced a recent crisis pregnancy. However, the descriptive statistics indicate that the percentage of married women who have experienced a recent crisis pregnancy has increased from 3.0% in 2003 to 7.4% in 2010, whereas the perecentage of cohabiting women and women in a steady relationship with this experience has decreased from 2003 (12.3% and 9.8%, respectively) to 2010 (8.0% and 2.6%, respectively). Finally, women for whom religion was not important to them were over-represented in the sample of women who had a crisis pregnancy in 2010, compared to those women in 2006.

**Table 1:** **Descriptive statistics for those who experienced a recent crisis pregnancy as a proportion of the overall respondents across the three surveys**

	**ICCP-2003 (n = 1961)**	**ISSHR (2006) (n = 2979)**	**ICCP-2010 (n = 1562)**	**Chi square**
	**[Sample %] n (weighted %)**	**[Sample %] n (weighted %)**	**[Sample %] n (weighted %)**	***χ*** ^**2**^ **(df) (φ)**
**Crisis pregnancy**	[4.9] 97 (7.4)	[5.0] 150 (5.4)	[5.7] 89 (6.1)	n/s
**Current age**				
18-25	[30.8] 34 (10.3)	[33.2] 73 (8.0)	[24.0] 27 (8.2)	n/s
26-35	[36.3] 45 (8.4)	[30.1] 56 (5.8)	[42.2] 38 (5.2)	
34-45	[32.9] 18 (3.5)	[36.6] 21 (2.6)	[33.8] 24 (5.6)	
**Current social class**				
SC 1-2	[30.8] 29 (5.3)	[36.8] 54 (4.4)	[37.8] 24 (3.4)	32.28 (6) (0.31)*
SC 3-4	[30.9] 34 (8.5)	[32.1] 52 (5.4)	[34.9] 27 (6.7)	
SC 5-6	[24.4] 19 (7.8)	[22.9] 35 (7.1)	[11.1] 9 (4.0)	
SC 7	[13.9] 15 (8.9)	[8.3] 9 (4.4)	[16.2] 29 (12.3)	
**Current education level**				
Pre-leaving cert	[21.9] 17 (9.5)	[21.8] 23 (5.5)	[14.5] 16 (13.6)	23.71 (4) (0.27)*
Leaving cert	[33.9] 22 (5.6)	[50.8] 70 (5.7)	[29.8] 19 (6.3)	
Post-leaving cert	[44.2] 58 (7.7)	[27.4] 57 (4.6)	[55.7] 54 (3.9)	
**Current no. of children**				
No children	[46.5] 16 (3.2)	[44.3] 38 (2.9)	[47.0] 6 (0.8)	23.72 (4) (0.27)*
One child	[14.0] 37 (24.2)	[13.8] 66 (17.8)	[14.8] 30 (10.5)	
2 plus children	[39.5] 44 (6.5)	[41.9] 46 (3.8)	[38.2] 53 (10.7)	
**Current relationship status**				
Married	[37.5] 31 (3.0)	[42.0] 41 (2.8)	[44.0] 40 (7.4)	n/s
Cohabiting	[5.0] 14 (12.3)	[8.6] 25 (10.7)	[11.7] 17 (8.0)	
Steady relationship	[19.3] 20 (9.8)	[16.5] 38 (8.3)	[15.1] 8 (2.6)	
Causal or no rel.	[36.2] 32 (10.4)	[32.9] 46 (5.7)	[28.7] 24 (5.1)	
**Current religiosity**				
Important	[62.6] 51 (5.6)	[78.9] 109 (4.7)	[57.3] 41 (5.7)	19.12 (2) (0.24)*
Not important	[37.4] 45 (10.6)	[21.1] 41 (7.7)	[42.7] 48 (6.5)	

*Note.* Chi square analysis conducted on unweighted data. Sample n denotes the overall percentage in the full sample of female respondents. n = sample size. *χ* ^2^ = chi square statistic. df = degrees of freedom. Φ = Phi effect size. n/s denotes the difference was not significant. *The difference was significant at the 0.005 level.

### Factors associated with crisis pregnancies [2003–2010]

Table 2 presents the characteristics of only those respondents who experienced a recent crisis pregnancy. There were no significant differences found across the three survey years in terms of the age at which the crisis pregnancy was experienced. The descriptive statistics indicate that a large percentage of those who experienced a recent crisis pregnancy in 2003 were aged 15–19 years (25.0%) or 20–24 years (32.1%) at the time, whereas in 2010 those who experienced a crisis pregnancy were more or less evenly split across the five youngest age groups, with approximately 20% in each age group. No statistical differences were found across the 2003 and 2010 surveys in terms of the contraception used at the time of conception. In 2010, 58.2% of those who reported a recent crisis pregnancy stated that no contraception was used at the time of the crisis pregnancy and a further 24.1% reported using a low efficacy contraception type. Again, there were no differences found across the three years in the chosen outcome for the crisis pregnancy. Almost 74% of the respondents opted for parenthood and 23% opted for abortion as the outcome for the crisis pregnancy in 2010 (3% missing data). Of the 97 women who reported a recent crisis pregnancy in 2003, 16 (16%) experienced more than one crisis pregnancy. Twenty-four of the 150 women (16%) who reported a recent crisis pregnancy in 2006 experienced more than one crisis pregnancy. Finally, 27 of the 89 women (30%) who reported a recent crisis pregnancy in 2010, experienced more than one crisis pregnancy.

**Table 2:** **Descriptive statistics for the age, contraception and outcome variables for those who experienced a recent crisis pregnancy at the time of the crisis pregnancy across three time periods**

	**ICCP-2003 (n = 97)**	**ISSHR (2006) (n = 150)**	**ICCP-2010 (n = 89)**	**Chi square**
	**n (unweighted %) (weighted %)**	**n (unweighted %) (weighted %)**	**n (unweighted %) (weighted %)**	***χ*** ^**2**^ **(df) (φ)**
**Age at CP**				n/s
15-19 years	26 (26.8) (25.0)	27 (18.0) (22.1)	15 (16.9) (19.2)	
20-24 years	19 (19.6) (32.1)	57 (38.0) (33.2)	20 (22.5) (19.5)	
25-29 years	22 (22.7) (18.4)	25 (16.7) (13.0)	19 (21.3) (19.6)	
30-34 years	16 (16.5) (13.3)	18 (12.0) (12.3)	23 (25.8) (21.8)	
35 years and older	14 (14.4) (11.3)	23 (15.3) (19.4)	12 (13.5) (19.9)	
**Contraception used at CP** ^**a**^				n/s^b^
No contraception	49 (50.5) (50.3)	-	53 (59.6) (58.2)	
Low efficacy	28 (28.9) (26.0)	-	23 (25.8) (24.1)	
Medium efficacy	16 (16.5) (19.5)	-	11 (12.4) (14.9)	
High efficacy	2 (2.1) (0.9)	-	1 (1.1) (2.0)	
Missing	2 (2.1) (3.2)	-	1 (1.1) (0.8)	
**Outcomes**				n/s
Parenthood	69 (71.1) (64.3)	87 (58.0) (58.9)	62 (69.7) (73.9)	
Abortion	16 (16.5) (20.5)	29 (19.3) (19.0)	23 (25.8) (22.8)	
Missing	12 (12.4) (15.2)	34 (22.7) (22.1)	4 (4.5) (3.3)	

*Note.* Chi square analysis conducted on unweighted data. n = sample size. *χ* ^2^ = chi square statistic. df = degrees of freedom. Φ = Phi effect size. n/s denotes the difference was not significant, ^a^The ISSHR survey did not ask respondents about the contraception they used at the time of the crisis pregnancy, ^b^The high efficacy contraception variable was not included in this analysis as the expected count was less than 5 and therefore it violated one of the assumptions of the Chi^2^ test.

### Predictors of a crisis pregnancy [2010]

A two-step hierarchical logistic regression was carried out on the ICCP-2010 data to determine the past sexual history and the current social demographic profile of the respondents who reported having recently experienced a crisis pregnancy. The results of this regression are presented in Table 3. In the first step, the three sexual history variables; age at first sex (dichotomised as before or after 17 years), contraception used at first sex, and receipt of sex education while growing up were entered into the regression model. The results indicated that previous sexual history was a significant predictor of an experience of a recent experience of a crisis pregnancy. Those respondents who received sex education while growing up were significantly less likely to experience a crisis pregnancy compared with those respondents who received no sex education (OR = 0.50, 95% CI = 0.29 - 0.85). Those respondents who reported first sexual intercourse after the age of 17 were less likely to experience a crisis pregnancy (OR = 0.42, 95% CI = 0.25-0.70), compared with those who had first sexual intercourse aged 17 or before this age. Furthermore, those who used contraception during their first sexual intercourse were also less likely to report the experience of a crisis pregnancy compared with those respondents who reported that they did not use contraception during their first sexual intercourse (OR = 0.19, 95% CI = 0.12-0.32).

**Table 3:** **Weighted binary logistic regression analysis explaining the association between the experience of a recent crisis pregnancy and sexual health history, contraception access and demographic characteristics (n = 1273)**

**Respondent characteristics**	**SE**	**Adjusted OR (95% CI)**
**Step 1: Sexual health history**		
**Sex education received**		
No		1
Yes	0.27	0.50 (0.29-0.85)*
**Age at first sexual intercourse**		
17 years and younger		1
Over 17 years	0.26	0.42 (0.25-0.70)**
**Contraception used at first sexual intercourse**		
No		1
Yes	0.26	0.19 (0.12-0.32)***
**Step 2: Demographic variables**		
**Sex education received**		
No		1
Yes	0.27	0.57 (0.34-0.98)*
**Age at first sexual intercourse**		
17 years and younger		1
Over 17 years	0.26	0.65 (0.39-1.07)
**Contraception used at first sexual intercourse**		
No		1
Yes	0.26	0.21 (0.13-0.35)***
**Respondent location**		
Rural		1
Urban	0.25	0.91 (0.56-1.49)
**Respondent age**		
18-25 years	0.41	5.50 (2.46-12.30)***
26 - 35 years	0.32	1.92 (1.03-3.59)*
36-45 years		1
**Social class**		
Social class 1-2		1
Social class 3-4	0.33	1.40 (0.74-2.67)
Social class 5-6	0.50	1.11 (0.42-2.96)
Social class 7	0.41	2.65 (1.20-5.89)*
**Relationship status**		
Married		1
Cohabiting	0.40	0.54 (0.25-1.18)
Steady relationship	0.54	0.21 (0.07-0.59)**
Casual/no relationship	0.36	0.36 (0.18-0.73)**
**Education**		
Pre-leaving cert	0.35	2.29 (1.16-4.52)*
Leaving cert	0.30	1.00 (0.55-1.81)
Post leaving cert		1
**Free medical care**		
Full medical card	0.31	1.21 (0.66-2.22)
GP card only	0.48	1.37 (0.54-3.48)
No medical card		1
**Migrant status**		
Migrant after 16	0.34	0.99 (0.51-1.93)
Not a migrant after 16		1

*Note.* SE standard error, OR odds ratio, CI confidence interval. *p < .05; **p < .01; ***p < 0.001

In the second step, the current demographic variables of the respondent were included in the model (respondent location, age, social class, relationship status, education, access to free medical care and migrant status). A test of the full model containing all predictor variables against constant-only model was statistically significant, *χ*^2^(17,1218) = 114.27, *p* < 0.001, indicating that the model was able to distinguish between those who experienced a crisis pregnancy in the previous six years and those who never experienced a crisis pregnancy. In the final adjusted model receipt of sex education (OR = 0.57, 95% CI = 0.34-0.98), and contraception used at first sex (OR = 0.21, 95% CI = 0.13-0.35) significantly predicted the experiencing of a recent crisis pregnancy. Age at first sex no longer predicted crisis pregnancy. Younger women (18–25 years) were more likely to report having experienced a crisis pregnancy in the previous six years compared with women in the older age group (36–45 years) (OR = 5.50, 95% CI = 2.46-12.30) as were women in the middle age group (26–35 years) (OR = 1.92, 95% CI = 1.03-3.59). Respondents in social class 7 (unclassified or unemployed) were more likely to experience a crisis pregnancy compared with those in social classes 1 and 2 (OR = 2.65, 95% CI = 1.20-5.89). Furthermore, respondents who were in a steady relationship, (OR = 0.21, 95% CI = 0.07-0.59) and those who were in a casual or no relationship (OR = 0.36, 95% CI = 0.18-0.73) were less likely to have experienced a crisis pregnancy, compared to married women. Respondents with pre-Leaving Certificate education were more likely to experience a crisis pregnancy compared to those with post-Leaving Certificate Education (OR = 2.29, 95% CI = 1.16-4.52). Current access to free medical care, locality, and migrating to Ireland after 16 years were not significant predictors of the experience of a crisis pregnancy.

A regression analysis was also conducted on respondents who had *ever* experienced a crisis pregnancy using the same model. The results were broadly similar to those who experienced a recent crisis pregnancy; however there were some notable differences. For those who ever experienced a crisis pregnancy;

- Age at first sex was a significant predictor of a crisis pregnancy in the fully adjusted model.
- There was no difference between the 26 to 35 years age group and the older age group in their likelihood of ever experiencing a crisis pregnancy.
- There was no education level effect for those who ever experienced a crisis pregnancy.
- Respondents who were in receipt of a full medical card were more likely to ever experience a crisis pregnancy compared with those with no medical card.

## Discussion

The overall benefits of successful family planning highlights the importance of research which examines the prevalence of, and characteristics associated with, crisis pregnancies in a particular country. Thus the current study is important in a number of ways. Through the wealth of data from three nationally representative surveys, the current study provides information not only on the characteristics associated with a crisis pregnancy in Ireland in recent years, but also tracks changes in some characteristics over a number of years and highlights the importance of one's sexual health history as a protective factor against the experience of a crisis pregnancy. The current study found that the proportion of women aged 18–45 who experienced a recent crisis pregnancy had increased from 4.9% of the sample in 2003 to 5.7% in 2010. Although this rate is higher than the prevalence of 5.2% for unintended pregnancies in the US [6], it is difficult to compare these rates given the differences in terms and definitions used across the two jurisdictions.

Ireland has experienced many changes demographically and economically over the past decade. Ireland moved from a period of economic boom to a recession from 2003 to 2010. During the same period the demographic of the population of Ireland changed considerably. Inward migration trebled between 1996 and 2006 with much of the migrants coming from the 20 to 34 age group [25]. The turn in the economy meant that this inward migration levelled and outward migration began to increase in the late 2000s. In 2010, 76% of all births were to Irish mothers; 14.3% were to mothers from EU states; a further 8.3% were to non-EU nationality mothers [26]. In 2010, 34% of births were outside of marriage compared with 31% in 2001 and there was a trend for women having fewer children and having children at an older age (the age at birth of first child increased steadily from 27.1 in 1997 to 29.4 in 2010) [26], all of which suggests changes in the style and structure of family formation in Ireland. The proportion of the population receiving higher levels of education also increased over the time period from 1995 to 2010 [27]. Furthermore, although Ireland is an overwhelmingly Catholic country, with 84% of the population describing themselves as Roman Catholics in the 2011 Census [28], the strong influence of religion in society has arguably declined over the past decade [29]. It has been argued that the meaning of a crisis pregnancy has changed in Ireland in recent years as a crisis pregnancy no longer forces a woman into marriage as it once did, and the main priorities for women who seek to have children are secure economic circumstances and social support [18]. These changes may be reflected in those who experienced a recent crisis pregnancy from 2003 to 2010. For example, the findings indicate that a higher percentage of women in the older age group experienced a crisis pregnancy in 2010 than in 2003, although this difference was not statistically significant. Furthermore, more married women and less cohabiting women and women in a steady relationship reported a recent crisis pregnancy in 2010, compared with previous years. A higher percentage of women with two or more children reported a recent crisis pregnancy in 2010, compared to previous years. These findings could be interpreted as supporting the contention that crisis pregnancies have changed their meaning in Ireland, with increased emphasis being placed on one’s economic and social supports rather than marital status when considering parenthood [18]. Furthermore, women for whom religion was not important were overrepresented in the crisis pregnancy sample in 2010 compared with 2006, although this may be due differences in the terminology of the religiosity variable across the surveys, or it may be due to a general decline in religiosity among the wider Irish population during this time period [29]. These current findings may therefore reflect both economic and societal value changes in Irish society.

Results indicate that the disparities between the groups who experience a crisis pregnancy is not increasing in Ireland as has been found in the US [6], but rather crisis pregnancies are beginning to affect women in all socio-economic groups, although some groups continue to be at an increased risk. Results of the regression analysis from the 2010 survey indicate that, similar to previous studies in other jurisdictions [6,13], younger women and women with a lower level of educational achievement were more likely to have experienced a crisis pregnancy. Unlike previous studies which found that cohabiting women were at an increased risk of an unintended pregnancy [6], the current study found no difference between cohabiting and married women in their likelihood of experiencing a recent crisis pregnancy. However, compared with married women, women in a steady, casual or no relationship were less likely to experience a crisis pregnancy. There was no effect for migrant status on the likelihood of experiencing a crisis pregnancy. These results would suggest that public health planning for crisis pregnancies should continue to be aimed at all demographic groups with additional targeted interventions for these groups of women who are at a particular risk.

Another important finding is the role of sexual health history as a protective factor against the risk of a crisis pregnancy. Although previous studies have investigated the effect of age at first sex and sex education on contraception use and teenage pregnancies [20-23], there is little research on the influence of such factors on unintended or crisis pregnancies. The current results suggest that, after controlling for other important demographic factors, the receipt of sex education and the use of contraception at the time of first sex reduces the likelihood of having experienced a recent crisis pregnancy. This finding has important implications for public policy in the provision of sex education. Such education may act as an important strategy in reducing the prevalence of crisis pregnancies and may provide opportunities to empower young women to feel they have a choice in whether and when they become pregnant.

Similar to previous findings [8,14-16], use of no contraception and use of poor efficacy contraception methods were cited as the most common contraceptive methods used at the time of conception of the crisis pregnancy. As nearly sixty per cent of the respondents reported not using contraception at the time of conception of the crisis pregnancy, and lack of contraception use at first sex was a predictor of crisis pregnancy, the findings may suggest that one’s behaviour at first sex may remain consistent throughout one’s sexual life. Given that previous research has suggested that cost can play a role in access to contraceptives, and the current findings in terms of lack of effective contraceptive use, public health policy should look at the affordability and accessibility of contraceptives, particularly for those at an increased risk of experiencing a crisis pregnancy.

Overall, these findings suggest that public policy should particularly target the risk groups, younger women and those with lower level of education, and focus on the early sexual experiences of young women in an attempt to reduce the incidence of crisis pregnancies. The role of sex education, and the high prevalence of no or low efficacy contraceptive use among those women who experienced a crisis pregnancy, highlights the importance of sex education in targeting risky sexual behaviour throughout the lifespan. Furthermore continued education on effective contraception methods and improved access to these methods is required, particularly for those groups of women who have been found to be at an increased risk. This education should go beyond school based sex education, perhaps through community-based youth services, as individuals with lower levels of education are already at an increased risk for the experience of a crisis pregnancy.

It was also found that nearly 23% of those who experienced a crisis pregnancy in 2010 had an abortion (n = 23) and this figure was slightly higher than in previous years. However, given the high number of missing data from the earlier surveys, this finding must be interpreted cautiously. The percentage of women who chose parenthood as a result of their crisis pregnancy was 74% in the latest 2010 survey, and again this figure is higher than in previous years. The outcomes of crisis pregnancies in Ireland appear to be considerably different to that found globally where the rate for parenthood is less than that of induced abortions [1]. This is likely due to prohibitive abortion legislation in Ireland. Essentially, in Ireland, unless their life is at risk, a woman must travel to a different jurisdiction to have an abortion.

There were a number of limitations of the study that should be noted when interpreting the results. The sensitivity of the questionnaire items may have led to social desirability in reporting and a misrepresentation in the results. For example, women have been found to considerably underreport the number of abortions they have experienced in face-to-face sexual health surveys [30]. This might suggest that the prevalence of abortions cited in the current study may be an underestimate given the manner of data collection. Furthermore, although telephone surveys have many advantages, they are subject to coverage bias from non-telephone households [31]. This was addressed somewhat by the inclusion of mobile phones in the sampling method for the 2010 survey. Moreover, telephone interviewing has been found to provide the least accurate responses when compared with interactive voice recognition and web surveys [32]. Research also indicates that there are differences among demographic groups in how they respond to questionnaires, and these differences may exacerbate over time [33]. Non-responders tend to be young, non-married, have a lower level of education; and the discrepancy in education level has increased over time [33]. Therefore it is possible that the current findings that younger women and women with a lower level of educational achievement are at an increased risk for a crisis pregnancy may be an underestimation given the data collection methods used and these finding should be interpreted cautiously in light of this. In order to minimise these sampling effects, quota sampling and reweighting adjustments were carried out on the data. The survey also relied on retrospective accounts of the experience of crisis pregnancy. Thus the demographic variables pertain to the time of the survey and may have changed since the experience of the crisis pregnancy.

Additionally, there is a lack of data regarding the abortion variable such that it was not known whether the abortion was carried out for medical reasons or for an unwanted pregnancy, or where the abortion was carried out. While the term ‘crisis pregnancy’ has been adopted in Ireland to overcome the limitations of the term ‘unwanted’ or ‘unintended’ pregnancies, there are difficulties inherent in its subjective nature and it limits comparison with other jurisdictions. There were also some differences across the surveys in the wording of the religiosity variable and the coding of the social class variable. For example, in the 2010 survey, unemployed respondents were not asked about their previous employment and thus all unemployed respondents were classed as social class 7, whereas the earlier surveys classed the respondent according to their previous employment. Thus, the findings across the surveys for the social class variable and the religiosity variable may be solely due to the differences in the wording of questions between the surveys rather than any differences in these variables observed across time.

## Conclusion

Overall, the current study provides important information with regard to who has experienced a crisis pregnancy in Ireland. Some particular strengths of the study were the large, nationally representative data sets and the similarity across studies which allowed for comparisons to be made. The study is particularly valuable as it shows the changes in the demographics of those who have experienced a crisis pregnancy in Ireland over the past number of years, similar to what has been done in the US [6]. Overall, the results indicate that women who have received sex education and used contraception at first sex are less likely to experience a crisis pregnancy, and younger women and women with lower levels of education are more likely to experience a crisis pregnancy. Differences were found in the current study in terms of the prevalence for crisis pregnancies in Ireland as compared with US estimates for unintended pregnancies [6] and the disparities between the demographic groups does not appear to be increasing as it has been in the US [6]. A further difference from global trends was found in the outcomes of crisis pregnancies, with a much higher prevalence for parenthood compared to abortion in Ireland. Overall these findings would suggest the public health policy needs to be tailored to the Irish context, with emphasis given to sex education and contraception availability aimed at all socio-demographic groupings with particular targeted interventions for younger women and women with lower levels of education.

## Abbreviations

GP: General practitioner
ICCP-2003: Irish contraception and crisis pregnancy survey 2003
ISSHR: Irish study of sexual health and relationships
ICCP-2010: Irish contraception and crisis pregnancy survey 2010
RDD: Random digit dialling
SC: Social class
OR: Odds ratio
CI: Confidence interval
ROI: Republic of Ireland
*χ*^2^: Chi Square
df: Degrees of freedom
Φ: Phi effect size
n/s: Not significant
SE: Standard error
